# Biodegradable tracheal stents: our ten-year experience with adult patients

**DOI:** 10.1186/s12890-024-03057-y

**Published:** 2024-05-15

**Authors:** Ludek Stehlik, Debarya Guha, Sheetal Anandakumar, Alice Taskova, Martina Koziar Vasakova

**Affiliations:** 1 Department of Respiratory Medicine, 1st Faculty of Medicine Charles University, Thomayer University Hospital, Videnska 800, Prague 4, 140 59 Czech Republic; 2 Department of Thoracic Surgery, 3rd Faculty of Medicine Charles University, Thomayer University Hospital, Videnska 800, Prague 4, 140 59 Czech Republic

**Keywords:** Interventional pneumology, Stents, Tracheal stenosis, Bronchoscopy, Polydioxanone, Biodegradable

## Abstract

**Background:**

Biodegradable (BD) stents made from polydioxanone have been used only in human airways. These stents combine the advantages of classical tracheal stents, and fewer side effects are expected due to their biocompatibility and their time-limited presence in airways. However, new clinical consequences have arisen. Here, the authors share their experiences with BD stents for tracheal indications, focusing on their safety and efficacy.

**Methods:**

This was a retrospective review of a collected database of adult patients who underwent implantation of biodegradable tracheal stents between September 2013 and December 2022 at the Department of Respiratory Medicine of the 1st Faculty of Medicine in Prague and Thomayer University Hospital. The indications included functionally significant nonmalignant tracheal stenosis and tracheomalacia. Self-expandable, biodegradable, polydioxanone tracheal stents manufactured by ELLA-CS Ltd. (Hradec Kralove, Czech Republic) were implanted during rigid bronchoscopy under general anaesthesia. All patients were followed up in the department and were provided with the necessary care. The main efficacy and safety parameters and relationships were analysed using descriptive statistics and Fisher´s exact, Wilcoxon and Kruskal‒Wallis tests.

**Results:**

A total of 65 stents were implanted in 47 adult patients. During the first two months after implantation, when adequate function was expected, the stent was found to be effective in 26 out of 39 patients who completed this period (66.7%). The clinical effectiveness reached 89.7%, as early restenoses were mostly mild and necessitated treatment in only 4 patients. The frequencies of significant mucostasis, migration and granulation tissue growth were 2.6%, 7.5% and 23.1%, respectively, during this period. Thirty-four participants completed the half-year follow-up period after the first or second stent insertion, and some were followed up beyond this period. Poor control of symptoms, the development of restenosis and the need for interventions were characteristic of this period as the stents degraded. Twenty-two patients who experienced remodelling or stabilization of the tracheal lumen achieved a stent-free state. Seven patients underwent subsequent surgical treatment.

**Conclusions:**

BD stents are safe and provide adequate tracheal support until they begin to degrade. The use of BD stents necessitates close monitoring of patients and accurate treatment of possible restenosis.

**Trial registration:**

Based on project NT14146 – Biodegradable stents in the management of the large airways (2013–2015, MZ0/NT), registered on May 1, 2013, in the Research and Development and Innovation Information System of the Czech Republic and at ClinicalTrials.gov (reg. no. NCT02620319, December 2, 2015).

## Background

Stenting is an important therapeutic option for many patients with narrowing of the central airways. If the narrowing has a benign origin, the lifespan and quality of life of patients depend mainly on maintaining a sufficient lumen, which is the only life-threatening condition. The placement of permanent silicone stents or covered self-expandable metal stents (SEMSs) is associated with specific disadvantages and complications. The most frequent complications are stent migration, limitation of mucociliary transport with mucus plugging, granulation tissue formation, stent fracture, and subsequent damage to the respiratory tract. Symptoms related to these complications negatively influence patients’ quality of life. Given these limitations, the treatment method should be chosen carefully, and surgical correction is preferred if possible. Patients who are not candidates for surgery due to comorbidities, anatomically disadvantageous trachea or technical issues constitute the majority of the stent-suitable group. Insufficient spaciousness after dilation in less complicated stenoses, the likelihood of collapse, and uncertain immediate postprocedural development of narrowing are all causes of attempts to secure the airway with a stent. Temporary support with a stent is primarily considered in patients before tracheal surgery and/or with anticipated improvement after respiratory rehabilitation, which may allow weaning from mechanical ventilation or a tracheostomy tube.

To overcome the drawbacks of traditional types of stents, during the last two decades, biodegradable (BD) airway stents have been developed. These stents combine the advantages of silicone and covered SEMSs. Fewer side effects are expected, as stents provide only temporary support (spending limited time in the airways), and because the stent material is considered biocompatible, it is less likely to interfere with human tissues. Biodegradable stents made from polydioxanone are the only ones used in human airways [1]. Polydioxanone is a biodegradable polymer from the polyester family that is commonly used in absorbable sutures. It is degraded by hydrolysis of its ester bonds into harmless degradation products. In vitro tests demonstrated that the radial expansion force of the stents began to decrease 3 weeks postimplantation, and after 9 weeks, the radial force decreased to half of its original value. After 12 weeks, significant disruption of the fibres and stent destruction occurred. Similar results were produced by in vivo testing and confirmed by human implantation experiences [2–6].

In some centres, BD stents are being implanted for obstructive bronchial complications after lung transplantation, in children with airway stenosis, and in adult patients with different types of airway narrowing [1, 3–6]. To our knowledge, in 2013, we were the first to start implanting BD stents in adult patients with tracheal narrowing. This work is devoted to the results achieved to date in one centre, and our aim is to draw attention to their advantages and highlight their limits in clinical use.

## Methods

### Patients and study design

We present a retrospective review of our collected database of adult patients who underwent BD polydioxanone tracheal stent implantation between September 2013 and December 2022 at the Department of Respiratory Medicine of the 1st Faculty of Medicine in Prague and Thomayer University Hospital. All patients who received BD polydioxanone custom-made stents (ELLA-CS Ltd., Hradec Kralove, Czech Republic) were included in the study.

The indications for stent implantation were functionally significant nonmalignant tracheal stenosis and tracheomalacia. Every patient was reviewed by two interventional pulmonologists and a thoracic surgeon to determine the best approach, and only those who were not candidates for primary surgical treatment underwent stent implantation. Contraindications included the presence of aero-digestive communication and pregnancy. We focused on demonstrating the effectiveness and safety of this approach. Special attention was given to a 60-day period postimplantation, which was considered generally safe by both us and the manufacturer, i.e., the period of anticipated guaranteed mechanical efficiency of the stent. As there is no chance of longer persistence of the biodegradable stent in the airways, a six-month period after stent implantation, or a second implantation if there was more than one stent, was deemed sufficient to evaluate long-term effects. More than 2 stents could be implanted. For practical reasons, mainly due to the initial nature of the narrowing and the related evaluation of stent efficacy, only the first or second stent effects were assessed. When relevant, for the sake of completeness of the overview, essential data on the implantation of other (third and fourth) BD stents and data from patients with longer follow-up are mentioned.

Efficacy was defined as an improvement in tracheal narrowing and relief from symptoms. The monitored phenomena included immediate postprocedure improvement of tracheal narrowing, details related to the procedure, and postoperative outcomes. A correct endoscopic effect was defined as a lumen improvement of at least 2 degrees according to Freitag´s classification [7] lasting 60 days postimplantation with no breach of integrity detected. Cases that did not fulfil this definition were considered stent failures. Clinical effectiveness was related to the need for subsequent bronchoscopy intervention for restenosis. Improvement of symptoms was defined as the reduction or disappearance of dyspnoea, easier coughing, loss of stridor, etc. Weaning from the tracheostomy tube and/or ventilator was assessed separately in patients as appropriate. Safety parameters included periprocedural events and complications, the need for stent fixation, endoscopy-detected situations, and complications, as well as the development of new symptoms, such as dyspnoea, stridor, respiratory failure, coughing up of stent fibres, haemoptysis, worsening of cough, and difficult expectoration. For events requiring intervention, the need for acute treatment, i.e., treatment carried out within 3 days from the first contact with the physician, was evaluated.

The time to detection of the first signs of disintegration of the stent (onset of rapid stent degradation), as well as the time to detection of the loss of the mechanical efficiency of the stent, were recorded. The first observed event was coughing up of the stent fibres experienced by patients or disruption of the fibres or the mesh of the stent being noted during bronchoscopy. The second event was a recurrence of stenosis due to apparent stent degradation or significant loss of stent integrity, as observed via bronchoscopy. Demographic data, type of stenosis (narrowing), aetiology and severity were collected from the hospital information system database. The presence of a dynamic component of stenosis (localized tracheomalacia) or diffuse tracheomalacia was considered a separate factor influencing stent degradation and outcomes.

### Stents and implantation technique

We used self-expandable, biodegradable, polydioxanone tracheal stents manufactured by ELLA-CS Ltd. (Hradec Kralove, Czech Republic) with the following dimensions (mm) (diameter × length): 18 × 50, 18 × 70, 20 × 50 and 20 × 70. The choice of stent size was guided by the decision of the bronchoscopist. Before implantation, the stents were manually loaded into the dedicated application apparatus, which worked as a pull–back delivery device. The trachea was intubated with a rigid bronchoscope with an internal diameter of 8.5–11.0 mm (Karl Storz, Germany). Patients were placed under total intravenous (i.v.) anaesthesia with jet ventilation. The distance was measured using a flexible bronchoscope and, when possible, under direct visualization using a thin flexible bronchoscope or rigid optics, and the stent was advanced to the desired location. Fluoroscopy was not used. After insertion, the position of the stent was adjusted using rigid forceps, and stent balloon dilatation was performed to compress the mesh of the stent towards the tracheal mucosa and to allow for better stent reopening. If deemed necessary, a thoracic surgeon secured the stents via external (percutaneous) fixation [8].

### Postoperative management and follow-up

After insertion, patients were observed in the hospital for at least one day; nebulized salbutamol, ipratropium and ambroxol were administered; and i.v. methylprednisolone (40–80 mg) was administered at the discretion of the physician. The first follow-up bronchoscopy was performed during the first week after the procedure. Patients were equipped with an aerosol nebulizer with a recommendation for regular inhalation of strongly mineralized water (Vincentka) and/or ambroxol solution b.i.d. A short course of alpha-aescin (40 mg orally, t.i.d., two weeks) was recommended to mitigate concomitant oedema. Additional follow-ups were carried out on a monthly or as-needed basis.

### Evaluation and statistical methods

Descriptive statistics and graphical presentations were used to summarize the data. Categorical variables were calculated and are expressed as counts and percentages; their relationships were investigated using Fischer´s exact test. For continuous variables, means and standard deviations (SDs) were calculated; t tests were applied for normally distributed variables, while Wilcoxon tests were used for nonnormally distributed variables. For more than two groups, the Kruskal‒Wallis test was used. The associations between variables were measured by the Pearson correlation coefficient. All calculations were performed with the help of the statistical program JMP 16.2.0 (SAS Institute Inc.).

## Results

From August 2013 to December 2022, BD stents were implanted in 47 adult patients with benign tracheal narrowing. In this cohort, 26 (55,3%) were females, and the mean (SD) age was 62.7 (13.3) years. Thirteen patients underwent implantation of more than one stent: 9 patients received two consecutive stents, 3 patients received three consecutive stents, and 1 patient received four consecutive stents. Significant tracheal stenoses (including those with localized short-segmented tracheomalacia) was the indication for stenting in 42 patients. The aetiologies of the stenoses were posttracheotomy (32 pts.), postintubation (8 pts.), and postsurgical (2 pts.), with the upper third of the trachea being the most common location. Five patients experienced tracheomalacia, i.e., collapse of the bulk of the trachea (with possible bronchial involvement). The majority of implantations went smoothly and were followed by a substantial increase in lumen capacity. Of the 65 stenting procedures, 8 (12.3%) experienced complications. We recorded two cases of nonpenetrating tracheal lacerations; one case of suffocation, probably due to the shift of the main collapse point to the periphery with the need for immediate stent withdrawal; two cases of laryngeal oedema; one case of pulmonary oedema with the need for in-stent intubation and short mechanical ventilation; one case of difficult stent placement; and one case of insufficient reopening due to stiff stricture with in-procedural displacement and damage to the stent and subsequent arduous stent extraction. Dislocation of the stent occurred in two patients during the two days after the procedure, and the stents were returned to their original position and externally fixed. External fixation was performed at the physician´s discretion in 21 (31.8%) procedures, including the two dislocation procedures. Among the 47 patients, 8 (17.0%) did not complete the two-month postimplantation observational period with the stent in situ for any reason, or the data were not available (Table 1).

**Table 1:** Patients who did not complete the two-month postimplantation period; *n* = 8

*No. of patients*	*Cause*
2	Died due to comorbidities
1	Data lost from study, died due to comorbidities during the 3rd month postimplantation
1	Data lost from study, further information not known
1	Vocal cord paralysis on 33rd day postimplantation (multiple myeloma subsequently diagnosed), stent withdrawal, retracheotomy
1	Suffocation probably due to a shift of the collapse point in tracheobronchomalacia, immediate stent withdrawal
1	Inadequate reopening due to anatomy and low radial force, stent withdrawal, continued with tracheostomy tube
1	Stent migration detected on Day 3 after insertion, withdrawal with no replacement, application of CPAP overnight

**Fig. 1:**
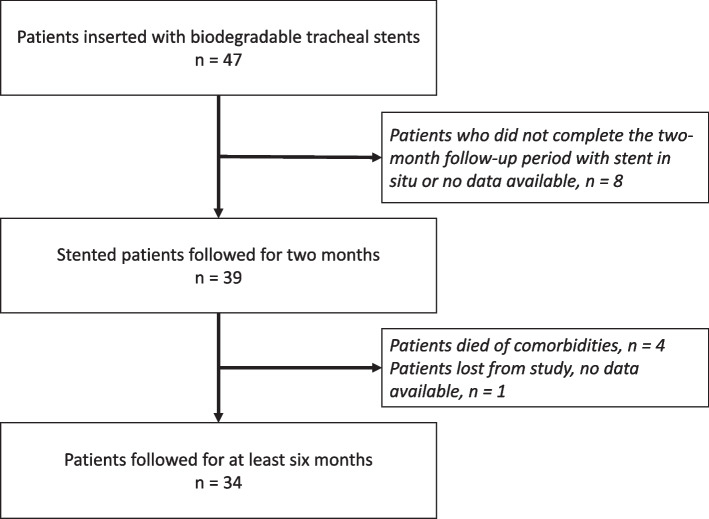
Flow chart of patients. The six-month follow-up period was considered after the first or second stent implantation. All patients with multiple stents completed this period

Thirty-nine patients (83%) completed the two-month follow-up (Fig. 1). During this period, 21 patients (53.8%) experienced uninterrupted relief of their symptoms. Stent efficacy, defined by the occurrence of its correct effect, was recorded in 26 patients (66.7%) in whom no (re)narrowing of the trachea was observed. The clinical effectiveness reached 89.7% in 35 out of 39 patients; most restenoses were not severe and did not necessitate bronchoscopy intervention, except for 4 patients (10.3%). Including early dislocations and severe restenosis after the first and second implantations, the probability of rigid rebronchoscopy in the two months after the procedure was 0.17 (9/53) for each stent placement (unsuccessful implantations, treatment failures due to other conditions and implantations of the 3rd and 4th stents were not included) (Tables 1 and 2). Table 3 summarizes the overall outcomes, symptom control and endoscopic features achieved during the 60 days after the first or second implantation in patients who completed this period.

**Table 2:** Stent failure in 13 patients

*No. of patients*	*Cause and solution*
1	Mild restenosis due to granulations, no intervention needed
3	Restenosis due to granulations, no intervention needed within 60 days postimplantation, balloon dilation performed later
1^a^	Restenosis due to granulations, balloon dilation and tissue removal; reoccurred with the second consecutive stent
2	Premature degradation of the stent, no further intervention
1	Premature degradation, no intervention within 60 days postimplantation, balloon dilation performed later
1^a^	Premature degradation, balloon dilation and removal of granulations
2^a^	Premature degradation, migration; removal of fibres and granulations, dilation, and BD restenting. Reoccurred with the second consecutive stent in one patient
2	Restenosis, no intervention during the 60 days postimplantation, tracheal resection performed later

^a^intervention needed within 60 days postimplantation

**Table 3:** Results in the first two months after implantation (*n* = 39)

Achievements		
- *uninterrupted relief of symptoms in 21 patients (53.8%)* - *correct effect of stents in 26 patients (66.7%)* - *no bronchoscopy intervention due to restenosis in 35 patients (*89.7%) - *decannulation possible immediately after or within a week in 13 out of the 15 patients dependent on a tracheostomy tube* - *weaning from ventilator and decannulation later in 1 patient simultaneously dependent on ventilatory support*		
**New symptoms**		
**Symptom**	**No. of patients**	**Treatment measures**
Purulent expectoration	3 (7.7%)	Antibiotics, mucolytics, intensified inhalations; extraordinary follow-up bronchoscopy in 8 patients; rigid re bronchoscopy 6 times in 4 patients. In 12 patients (30.8%), measures were introduced within 3 days
Dyspnoea	8 (20.5%)	
Difficult expectoration	3 (7.7%)	
Coughing up of fibres	2 (5.1%)	
Haemoptysis	1 (2.6%)	
Subglottic oedema and fibrin plaques postimplantation	2 (5.1%)	Short-term mechanical ventilation/noninvasive ventilation
**Endoscopic findings**		
**Finding**	**No. of patients**	**Treatment measures**
Mucosal hyperplasia (in the stented tracheal segment), locally increased serous secretion	all	None
Secretions in larger quantities in the stented section and peripheral airways	1 (2.6%)	Mucus removal in a patient with severely impaired ability of expectoration
Granulation tissue overgrowth with some narrowing of the lumen	9 (23.1%)	Mechanical removal and balloon dilation two times in 1 patient
Stent migration (early)	2 (5.1%)^a^	Stents returned to original position and externally fixed
Premature degradation	6 (15.4%)	Removal of fibres, dilation and BD restenting in 2 patients (twice in one patient); dilation and granulation removal in 1 patient

^a^One additional dislocation occurred; the patient is not included in the table

Thirty-four patients were followed up for at least six months after implantation of the first and only stent (21 patients) or second BD stent (13 patients). Table 4 shows the data from the follow-up period from the beginning of the third month after implantation. The mean time to onset of rapid stent degradation was 77.4 (14.9) days, and the estimated time to complete loss of the mechanical properties of the stent was 84 (19.7) days. Both periods were shortened by premature degradation, with the implanted stents nearing the end of the manufacturer's declared expiration time (3 years at that time). After our notification, the manufacturer admitted that deviations in the process of stent degradation could be caused by the length of storage, and the expiration date was reduced to one year. Extraoesophageal reflux may have been the cause in the sixth patient. In 13 patients who needed second stents, the mean time to restenting was 89.2 (27.4) days after the first implantation.

**Table 4:** Results from the third month after implantation (*n* = 34)

Achievements		
-* uninterrupted relief of symptoms in 6 patients (17.6%)* -* remodelling of the stenosis or stabilization of tracheomalacia in 22 patients (64.7%)* -* patients benefiting from treatment: 31 (91.2%)*		
**New symptoms**		
**Symptom**	**No. of patients**	**Treatment measures**
Dyspnoea	17 (50.0%)	Bronchoscopy intervention and complex care
Difficult expectoration	17 (50.0%)	
Coughing up of fibres	16 (47.1%)	
Haemoptysis	1(2.9%)	
Severe dyspnoea, asphyxia after hospital admission	1 (2.9%)	Successful resuscitation, restenting, occurred on day 88 after implantation, patient noncompliance
**Endoscopic findings**		
**Finding**	**No. of patients**	**Treatment measures**
Mucosal hyperplasia (in the stented tracheal segment), locally increased serous secretion; decreasing intensity from the 3rd month	All	None
Significant granulation tissue growth	16 (47.1%)	See below
Renarrowing accompanying stent degradation with or without the contribution of granulations	28 (82.4%)	Additional interventions in 28 patients (82.0%): balloon dilation + removal of granulation (without restenting) in 16 patients (47.1%), twice in two of them, with a mean time of 93.6 (25.9) days from insertion; second stents implanted in 13 patients (38.2%) with a mean time (SD) to the first implantation of 89.2 (27.4) days; third stents in three patients; fourth stent in one; for final outcomes, switches to permanent stents and surgical treatment, see Fig. 2
Larger stent remnants in the trachea	8 (23.5%)	After consideration, removal in 4 patients, using the rigid technique simultaneously with the treatment of restenosis in 2 patients
Stent fibres stuck in the left main bronchus	1 (2.9%)	Removal using flexible technique

**Fig. 2:**
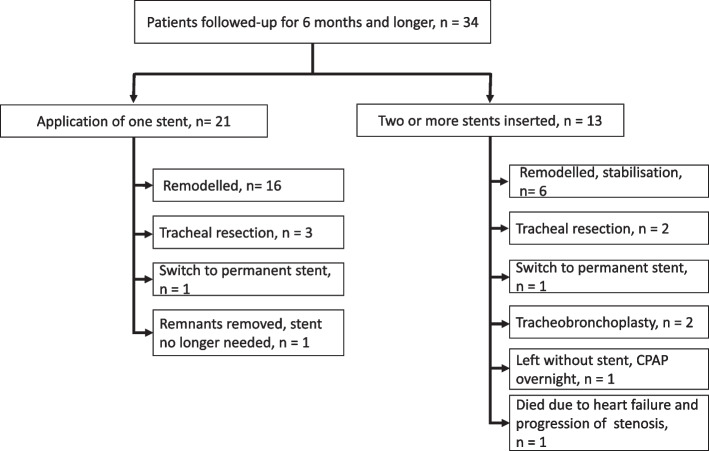
Results of long-term patient monitoring

Twenty-two out of the 34 patients experienced remodelling of stenosis or stabilization of tracheomalacia, i.e., a condition where they no longer needed mechanical support of the trachea or any other treatment, within a mean (SD) of 5.9 (2.8) months since the first implantation. For patients who were treated with one to two stents, the longest time to remodelling/stabilization was 8 months. In two patients who had three stents implantation, remodelling/stabilization occurred by 10 and 14 months after the first implantation. The last patient in whom three stents were implanted continued with intermittent continuous positive airway pressure (CPAP) due to residual dynamic narrowing. No remodelling occurred in the patient in whom four BD stents were implanted; the patient was sent for tracheal resection. Similarly, other solutions were chosen to maintain airway patency in the remaining patients. One patient whose trachea was stented due to pressure caused by a temporarily inserted oesophageal stent (for a fistula in the anastomosis after oesophageal resection) achieved good effects but could not be included in the remodelling group (Fig. 2).

In total, 84 bronchoscopic interventions were performed in long-term monitored patients (i.e., 2.47 interventions per patient), including procedures in the first 2 months after stent insertion. Among these, there were 52 insertions of BD stents and 2 insertions of permanent stents; the rest were nonstenting interventions, most of which (except for 3) were performed using a rigid technique. The probability of subsequent intervention (including the introduction of another BD stent) for the first implanted BD stent was 0.68 (34/50). Table 5 shows the baseline patient characteristics and final outcomes.

**Table 5:** Baseline patient characteristics and final outcomes

**Patient**	**Sex**	**Age**	**Aetiology of narrowing**	**Degree**^a^	**BD stents used**	**No. of other procedures**	**Results**
1	M	60	PTTS	32000	1	0	Remodelling
2	F	52	PITS	13000	1	1	Remodelling
3	M	74	TM, after correction of a. lusoria	23322	2	0	Stabilization, weaned from ventilator
4	M	59	PTTS	30000	2	0	Remodelling
5	M	58	PITS	00200	1	0	Remodelling
6	M	67	PTTS	13000	3	2	Remodelling
7	M	29	TM (congenital)	32111	2	0	Stabilization, decannulation after 8 months
8	M	45	PITS	30000	1	1	Remodelling
9	M	66	PTTS	30000	2	1	Residual narrowing, died due to other causes
10	F	65	TM (COPD)	31000	1	1	Improved, subsequent tracheoplasty
11	F	74	PTTS	02000	1	0	Died due to heart failure
12	M	58	PITS	04000	2	1	Restenosis, switch to permanent stent
13	M	61	PITS	04000	1	1	Remodelling
14	F	63	PTTS	04000	1	1	Died due to heart failure
15	M	37	Postsurgical, after tracheal resection	02200	1	2	Remodelling
16	F	72	PITS	20000	2	0	Remodelling
17	F	74	PTTS	20000	1	0	Died due to heart failure
18	F	76	TM (COPD)	14222	2	1	Improved, subsequent tracheoplasty
19	M	37	PTTS	42000	1	1	Residual narrowing, tracheal resection
20	F	68	PTTS	30000	1	1	Died due to comorbidities
21	F	83	PITS	30000	1	0	Remodelling
22	F	70	PTTS + TM	30000	1	0	Data lost from study
23	F	85	PTTS	30000	1	1	Died due to comorbidities
24	M	63	PTTS	30000	4	1	Restenosis, tracheal resection
25	F	59	PTTS	30000	1	0	Remodelling
26	F	62	PTTS	30000	3	1	Remodelling with slight residual narrowing
27	F	77	PTTS	24422	1	0	Data lost from study
28	F	62	PTTS	30000	1	0	Remodelling
29	M	70	PTTS	20000	1	1	Remodelling
30	F	71	PTTS	20000	1	0	Remodelling
31	F	43	PTTS	30000	1	1	Remodelling with some residual narrowing
32	M	78	PTTS	30000	1	1	Vocal cord paralysis on 33rd day postimplantation, multiple myeloma, stent withdrawal, re-TS
33	F	57	TM (congenital)	04433	1	0	Suffocation due to shift of collapse point, unsuccessful insertion, immediate stent withdrawal
34	F	55	PTTS	40000	2	3	Restenosis, tracheal resection
35	F	61	PTTS	32200	1	1	Restenosis, permanent stent
36	F	77	PTTS	20000	1	1	Remodelling
37	M	72	PTTS	30000	1	0	Remodelling
38	M	72	Postsurgical, compression by an oesophageal stent for an anastomotic leak	03310	1	1	Remnants removed; stent no longer needed as anatomical conditions improved
39	F	48	PITS	20000	1	1	Residual narrowing, tracheal resection
40	F	58	PTTS	30000	3	0	Residual narrowing, continued with CPAP overnight
41	M	24	PTTS	10300	1	2	Remodelling
42	F	79	PTTS	32000	1	0	Inadequate reopening due to low expansion force, stent withdrawal, continued with tracheostomy tube
43	F	71	PTTS	20000	1	1	Early migration, left without stent (refused reimplantation) continued with CPAP overnight
44	M	62	PTTS	31000	2	1	Died due to heart failure and progression of stenosis
45	F	63	PTTS	32200	1	1	Remodelling
46	M	73	PTTS	30000	1	0	Died due to comorbidities
47	M	63	PTTS	30000	1	1	Residual narrowing, tracheal resection

*PTTS* posttracheostomy tracheal stenosis, *PITS* postintubation tracheal stenosis, *TM* tracheomalacia, *COPD* chronic obstructive pulmonary disease, *TS* tracheostomy, *CPAP* continuous positive airway pressure

^a^According to Freitag et al. A proposed classification system for central airway stenosis (2007), Code 0 is assigned to nonappreciable stenosis, and codes 1, 2, 3 and 4 are assigned to ∼25, 50, 75 and 90% decreases in cross-sectional area, numerical positions: upper-mid-lower trachea-right main bronchus-left main bronchus

During the first two months after implantation, the onset of new symptoms or their repeated worsening were more frequent in patients with more severe initial narrowing, i.e., a reduction in lumen capacity of 75% or more (p = 0.0267). The presence of a malacic component in the narrowing could be related to the achievement of the correct stent effect. However, a larger group of patients would be required to demonstrate this dependence (p = 0.0905). Stents started to disintegrate earlier in the malacic component group: 75.5 (13.9) vs. 82.2 (16.3) days (SD); *p* < 0.0001. The abovementioned premature degradation did not influence the results. Other clinically relevant analyses were not significant.

## Discussion

### Implantation and early management

**Fig. 3:**
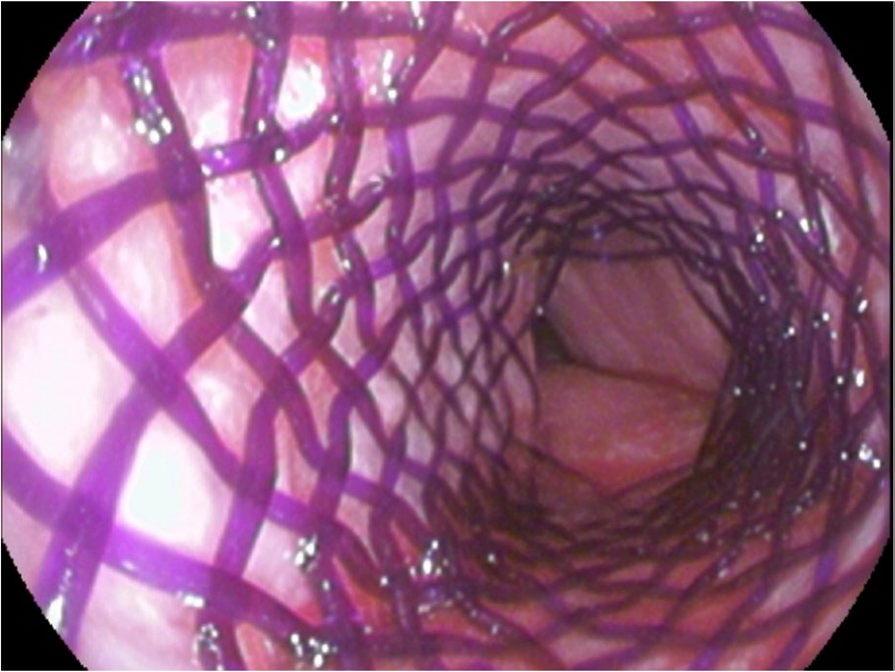
Stent in the trachea immediately after implantation

**Fig. 4:**
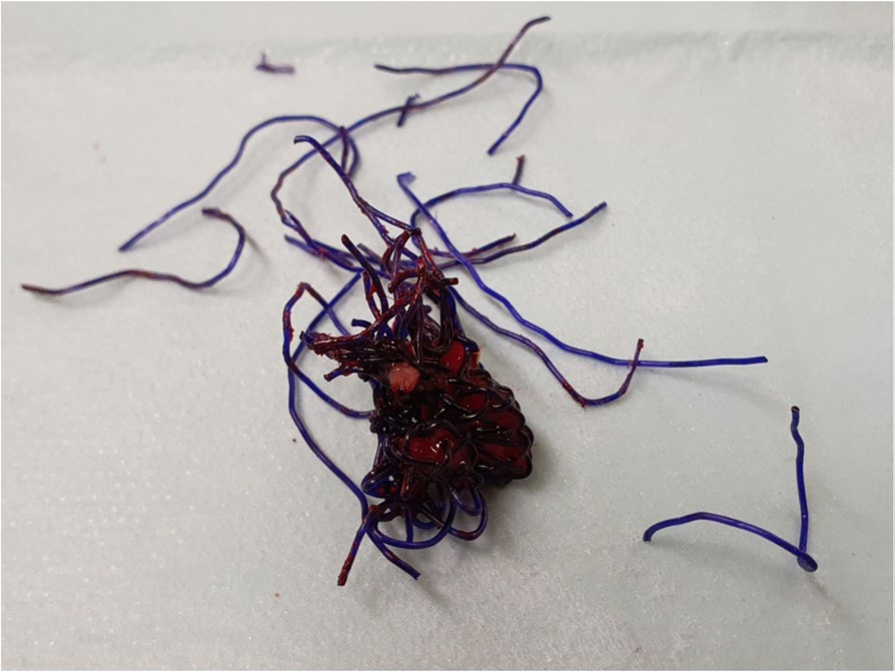
Deformation of the stent caused by extraction. During the implantation procedure, this stent migrated under the stenosis and became deformed during its extraction. Rigid endoscopic scissors and forceps had to be used

The majority of stent implantations went smoothly and were followed by a substantial increase in lumen capacity. The stents showed good adaptation to the airway anatomy immediately after insertion (Fig. 3). Of the 65 stenting procedures, 8 (12,3%) were accompanied by difficulties mostly related to the rigid technique or the general condition of the patients. The technical difficulty of placement seemed to be somewhat lower than that of silicone stents, which are slightly more complex, or almost comparable to that of SEMSs. Rigid bronchoscopy and general anaesthesia are necessary for tracheal application. Some SEMSs can be introduced with a flexible technique, which is a theoretical advantage [9]. Insufficient reopening due to a stiff stricture in patients is unlikely to be serious and should not be considered a complication, unless it is followed by distal migration of the stent (under the stenosis). In this case, extraction of the stent was subsequently problematic because the stent was completely deformed when it was pulled out with rigid forceps (Fig. 4). This issue appears to be typical of BD stents since they do not have any extraction threads and may deform in a disorganized manner during manipulation. To our knowledge, we are the first to report this complication with BD stents. Although the integrity of the polydioxanone stent and its fibres is pronounced at the beginning, the radial force itself is less than that of SEMSs. Therefore, thorough balloon dilatation or further additional treatment of the lumen before stent insertion is a prerequisite.

External fixation was performed in 21 (31.8%) implantations and, except in two patients, was performed immediately after deployment of the stent. External fixation appears to be a suitable supplement to implantation, especially for stents in the upper trachea and in cases of posttracheotomy stenoses, where there is a greater risk of migration [8, 10, 11]. Although it prolongs the procedure and makes the implantation more difficult, the stent does not have to produce a significant radial expansion force, which does not allow dislocation but does lead to mucosal irritation. The technique we used was similar to that used for permanent stents [8, 12]. Over the years, as we gained experience, we used it less often. The securing stitch was always removed after 2 to 3 weeks, so its impact on the patients' quality of life and aesthetics was minimal.

### Stent migration

In the case of BD stents, we can distinguish between early dislocation when fibres of the stent are not incorporated in the mucosa and late dislocation, which involves pieces of mesh and fibres and a period of stent degradation. Early dislocation occurred in 4 patients—4 events (6.5%) out of 62 successfully implanted stents. In two patients, the stents were repositioned and fixed, while the third patient refused this procedure and was satisfied with overnight CPAP after stent removal. In the last patient, the stent moved 10 mm distally, and no intervention was necessary. Although we considered not securing the stents immediately after implantation because of our lack of experience at that time, we did not classify early stent dislocations as failures. The repositioned stents worked well afterwards, and their properties were not affected. In benign stenoses, we encountered a relatively large number of stent migrations, at a frequency of approximately 20% [11]. This incidence has been previously described to be greater, especially in cases of upper tracheal narrowing treated with either silicone stents or SEMSs [10, 13–16]. Dumon et al. reported a frequency of migrations in 18.6% of all silicone stents inserted for benign tracheal stenosis [17]. However, with a newer type of silicone stent with narrow central and widened distal portions, which was intended for benign tracheal stenosis, there was no stent migration at a mean follow-up time of almost 23 months [18]. The low number of stent migrations recorded in this study is likely because BD stents are uncovered and adapt well to the anatomy of the airways and to the use of external stent fixation.

During stent disintegration, parts of the stent (mesh or fibres) may break off and move distally to the periphery. In two patients, this occurrence was detected during premature degradation. In another patient with a reduced ability to expectorate, the fibre pieces were stuck in the left main bronchus. The authors agree that in children, these events may be dangerous due to their smaller airway dimensions [19].

### Mucostasis and related features

In classic stents, mucostasis is the most common side effect and is observed in at least one-third of all stent patients [11]. In our cohort, only one case of narrowing due to mucus stagnation was observed in a patient with a strongly impaired expectoration ability (1 of 39; 2.6%). Dumon et al. reported a mucostasis frequency of 5.7% [17] for silicone stents used for tracheal stenosis; Brichet et al. reported mucostasis in 5 of 18 patients stented due to postintubation tracheal stenosis [20]; and Karush and colleagues identified mucus accumulation as the most frequent cause of reintervention in patients with silicone stents placed for benign central airway stenosis (60%, 131/220) [14]. Sputum retention was significantly greater for both noncovered and covered modern SEMSs (15% and 42%, respectively) [21], and a frequency of 15% was reported by Dahlquist et al. for fully covered SEMSs, while Fortin et al. reported a frequency of only 2.5% [15, 16]. Mucostasis can be associated with halitosis resulting from colonization of the stent with bacteria and fungi [11]. None of our patients complained of this condition.

Subjective intolerance was observed in 5% of patients treated with fully covered SEMSs by Fortin et al. [16]. Xiong et al. reported cough and chest pain in 25% and 21% of patients, respectively, who were implanted with SEMSs; lower rates were observed for bare stents [21]. Haemoptysis occurred slightly more often with bare SEMSs than with covered SEMSs: 17% vs. 12%, respectively. In patients with silicone stents, the occurrence of haemoptysis is usually linked to the stent introduction and related trauma of the airways [17]. In our study, pain or discomfort related to the expansion force of the stent was not reported by any patient, while purulent and difficult expectorations were reported by 15% of patients during the first two months postimplantation and in 50% of patients thereafter when they experienced stent degradation. Only one patient reported haemoptysis, which was related to the repeated irritation of the mucosa due to contact with the loose portion of the stent in the unconstricted part of the trachea.

### Granulation tissue formation

**Fig. 5:**
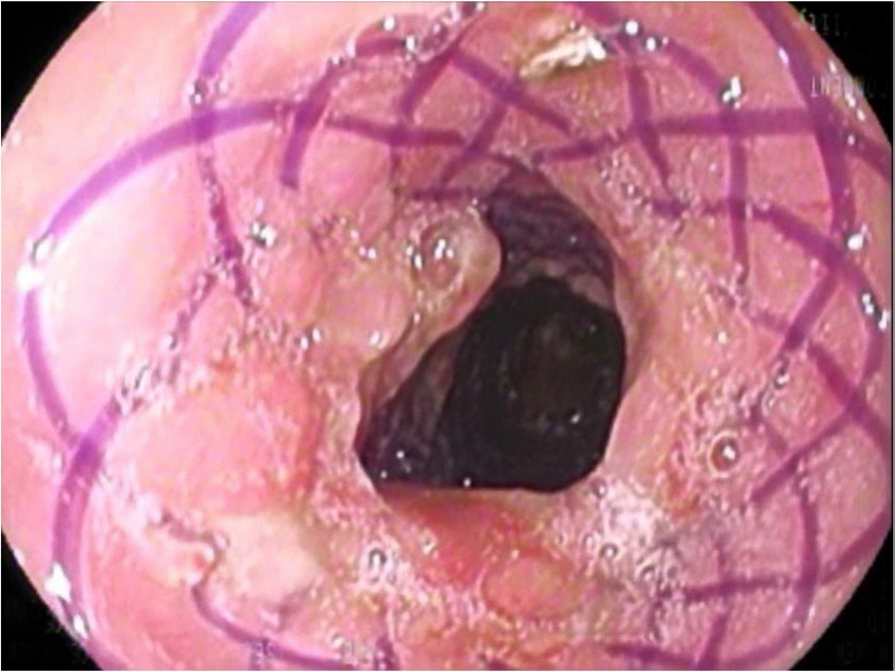
Granulation tissue formation one month after implantation. This finding is most pronounced in the place of the original largest narrowing. Serous hypersecretion

The inflammatory mucosal response is an important determinant of stenting success. In animal studies, necrosis of the epithelium and lamina propria at the point of contact between the stent fibre and the mucosa has been proven. Hyperplasia of goblet cells resulting in hypersecretion was also observed [2]. Valverde et al. [22] showed that successive stenting caused mild inflammatory changes in the tracheal wall and no increase in the collagen matrix or modifications in the cartilage structure. Generally, the response to damage is the formation of granulation tissue. In an animal study conducted by Choi and colleagues, granulation tissue growth occurred mainly beside stent fibres, reaching a maximum at 4 weeks and decreasing in thickness at 8 weeks postimplantation [23]. Rodriges Zapater et al. [24] reported that the overall granulation formation rate was 30% in their study of rabbits. In our study, significant granulation tissue growth with some narrowing of the lumen was detected in 9 out of 39 (23%) patients during the first two months postimplantation and in 16 out of 34 (47%) patients after the first two months. The contribution of this growth to renarrowing was difficult to assess in cases of concomitant stent degradation. In all patients, we exclusively used stents with a diameter of 18 or 20 mm, which could have played a role in the induction of granulations in patients with differently narrowed tracheas (Fig. 5).

Granulation tissue growth occurs to some degree in more than half of all patients; in approximately one-quarter of all patients, it becomes clinically significant [11]. Low complication rates were reported for some covered SEMSs (7.5%) [16]; however, other authors reported an incidence of up to 35% [15]. Granulomas induced by excessive mobility were reported in 17.2% of all silicone stents inserted for benign tracheal stenosis by Dumon and colleagues [17] and in only one of 13 patients treated with a silicone stent with a narrowed central part [18]. For uncovered SEMSs, granulation tissue growth (through the mesh of the stent) is generally more pronounced, making their extraction difficult, so they are not usually the first choice for benign stenoses.

### Weaning from tracheostomy cannulas

Our stents allowed a subgroup of 14 patients to undergo removal of the tracheostomy tube or weaning from the ventilator. We thus build on the comprehensive work of Noppen et al., who focused on permanent stents to address this issue [25]. Evidence of the suitability of BD stents is supported by experience in both children and adults [5, 6, 19, 26]. We and other authors used a temporary combination of a tracheostomy cannula placed with its end in the stented section of the trachea to secure the airways [5, 6]. We mention this patient group separately to highlight one of the possible indications for BD stents. These patients were in very poor general condition and often had a poor ability to expectorate; therefore, from our point of view, they were unlikely candidates for a permanent stent. The removal of the tracheostomy tube improved their psychological state and enabled further progress in their rehabilitation.

### Stent degradation

**Fig. 6:**
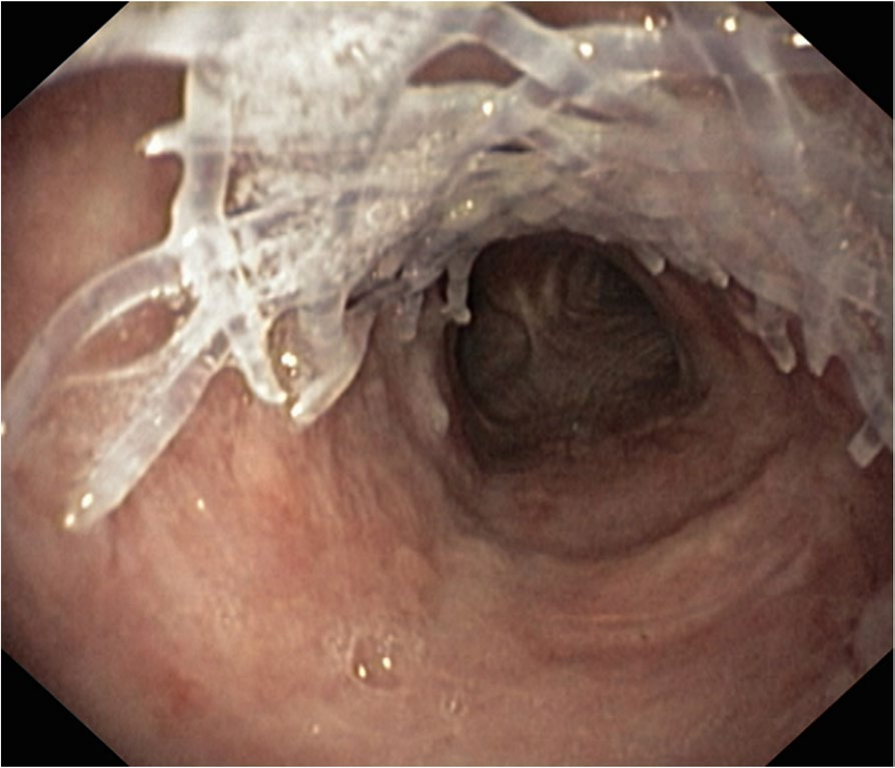
Irregular degradation of the BD stent three months after implantation. The missing piece of the stent was expelled by the patient

At the onset of rapid degradation, stents quickly lose their mechanical properties and disintegrate. This period begins with the occurrence of a fracture in the first stent fibre and technically ends with the complete loss of the supporting capabilities. Even beyond this point, individual stent fibres can subsequently be found to be embedded in the hyperplastic mucosa. Some of the fibres are coughed up by the patient, and some are absorbed by the airway wall. Novotny et al. suggested [2] that swallowing and ingestion could have occurred in addition to expectoration. In rabbits sacrificed at week 10 after implantation, no stent material was found. Morante-Valverde and colleagues [22] observed complete degradation by the 14th week; Choi et al. [23], who worked with polydioxanone stents produced elsewhere, reported that the stent disappeared by the 12th week. The latter study assessed whether the fragments had entered the lungs, but no fragments were found. Unlike this animal experiment, we had to address dislocation of the bundle of fibres into the left main bronchus in one patient with impaired expectoration. Similar experience in a child was reported by Sztanó and colleagues [19], who considered unpredictable and nonlinear degradation to be a substantial disadvantage. The process of stent disappearance and its consequences in humans have been well described by clinicians [3–6, 19]. Based on our observations, the predominant elimination pathway seems to be mostly determined by the apposition of the stent to the wall of the airways. The parts of the stent without close contact with the wall degraded first (Fig. 6). Our stents started to disintegrate earlier in patients with localized or extensive tracheomalacia (75.5 (13.9) vs. 82.2 (16.3) days (SD); *p* < 0.0001), which could be due to repeated mechanical stress on the stent. This observation agrees with the work of Choi et al. [23].

### Long-term results and clinical consequences

**Fig. 7:**
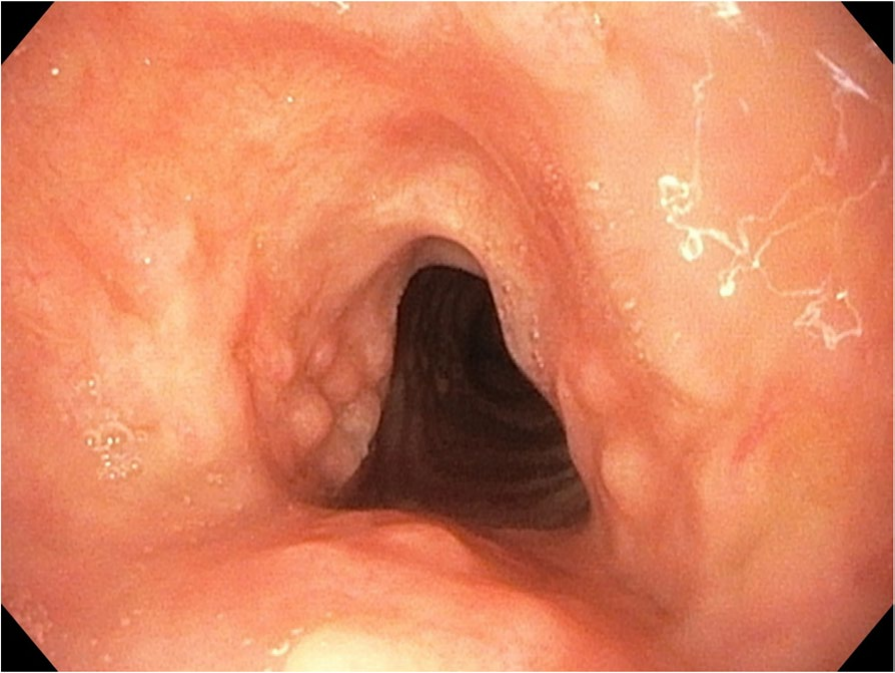
Remodelled upper tracheal narrowing six months after implantation. Islets of hyperplastic mucosa represent an imprint of a degraded stent

We achieved a “without stent” state in 22 out of the 34 patients. The mean (SD) time to cure was 6 (3) months, and the longest was 14 months. In addition to the fact that the stents themselves were responsible for tracheal remodelling, balloon dilations within and outside stenting procedures could have been beneficial (Fig. 7). The use of a combination of stenting and nonstenting techniques is common in postintubation and posttracheostomy stenoses and leads to permanent improvement in lumen spaciousness [27]. After degradation in our tracheomalacia patients, we did not observe reduced collapsibility of the lumen compared to the original state, which was contrary to our original assumptions [6]. We judged that improvements in the general condition of patients played a vital role. Dutau et al. reported that no recurrence after stent removal varied from 40% when a permanent stent was placed for 6 months to 70% when it remained in place for 18 months [28]. The time required to heal the stenosis thus appears to be longer. Galluccio et al., who used silicone stents in some simple stenoses and all complex stenoses, reported a mean duration of stenting of 18 months; in the case of complex stenoses, the mean duration of treatment (SD) was 20 (3) months [29]. With this method, they were able to cure 69% of complex lesions. Brichet and colleagues were less successful in this regard, but their treatment algorithm favoured stent extraction after 6 months in patients who became candidates for surgical treatment [20]. The BD stents were characterized by very good patient tolerance and safety within the first two months after implantation, but worse symptom control occurred starting in the third month. The safety of the stents in this period depends mainly on care management. We honoured the patient’s preference for surgical treatment, which unfortunately was not initially feasible for any of our patients. However, because of the sufficiently long periods of relief needed for rehabilitation, surgery could be performed later in seven of these patients. With BD stents, there is no need to consider the time of stent extraction due to their limited lifespan. Instead, the physician is faced with the decision of whether to dilate, restent, or switch to another treatment option. In our study, we detected an average of 2.47 therapeutic bronchoscopies per patient in the long-term monitoring group. Galluccio et al. reported a mean of 2.07 procedures in patients with simple stenosis and 3.27 in those with complex stenosis, using silicone stents in the latter cohort [29]. Sun et al. reported the average need for 4 or 5 procedures for successfully and unsuccessfully treated patients using a variety of procedures, including stents [27]. Focusing on third-generation SEMSs, Fortin et al. reported the need for more than two bronchoscopy interventions per patient [16]. Therefore, we believe that biodegradability can reduce treatment time while maintaining the number of necessary interventions at an acceptable level. Table 6 shows the positions of BD stents and permanent stents for various tracheal indications.

**Table 6:** Basic characteristics of biodegradable and main types of permanent stents

	**Indication**	**Implantation**	**Advantages**	**Drawbacks and complications**
Biodegradable stent	Benign narrowing Authors suggest: posttracheotomy and postintubation stenosis, weaning from tracheostomy cannula	General anaesthesia, rigid technique for tracheal application, ease of deployment similar to some SEMS (pull-back delivery device), may require external fixation	Very good adaptation to the airway anatomy, low migration rate (6.5%), excellent tolerance and minimal mucostasis, shortening of stenting time	May deform during repositioning, granulation tissue formation: 23%—47% (early—late period), poor symptom control and restenosis common from the 3rd month onwards, unpleasant expectoration of fibres (50% of patients), dislocation of stent parts during degradation
Silicone stent	Benign and malignant	General anaesthesia	Good tolerance, can be adjusted on site, easy to remove, well established and safe in benign indication	Migration rate 18,6%—33.3%, granuloma formation up to 17,2% in benign tracheal stenosis (but no and minimal in stenotic stents); impaired transport of secretions and mucus plugging 5,7%—60%; bacterial colonization and halitosis
SEMS	Malignant, benign narrowing with newer SEMS	General anaesthesia preferred	Easy to insert, possibly with flexible bronchoscope (if rigid is not available), high expansion radial force	Migration rate 11,9%—32,5%, granuloma formation 7,5%—35%, sputum retention 2,5% -42,4% for fully covered 3rd generation stents in benign tracheal stenosis; for uncovered: 1,4%, 4,2% and 18,1% as probably the best reported values. Possible airway perforation, stent rupture, difficult extraction (depending on design)

### Study limitations

Our study had several limitations. This was a retrospective review, and the set of evaluated patients was still small and heterogeneous, including patients with different aetiologies and types of tracheal narrowing. The symptoms assessed were not quantified, and a quality-of-life assessment was not used. Endoscopic evaluation of airway dimensions was limited, and the study was conducted at a single institution. In the analysis of the patient group, the most clinically relevant phenomena and categories from the authors' point of view were considered, with follow-up duration as a covariate.

## Conclusions

Biodegradable tracheal stents are relatively novel types of lumen-supporting devices. In our group of adult patients, they temporarily provided satisfactory and efficient compensation for tracheal narrowing in most cases. In some patients, these stents also enabled complete healing of the narrowing, weaning from the tracheostomy tube, or the definitive surgical treatment of tracheal narrowing.

Stents are easy to insert, adapt well to the anatomy of narrowed airways and can be secured by external fixation to prevent migration. Therefore, they may be appropriate for posttracheotomy stenoses when tracheal resection cannot be performed primarily. BD stents minimally limit the transport of secretions through the respiratory tract. However, they induce the growth of granulation tissue, and specific difficulties are associated with their decreasing mechanical properties due to degradation. Once implanted, patients must be closely followed up, and the physician must have a plan for how to address restenosis.

## Data Availability

The datasets supporting and supplementing the conclusions of this article as well as some additional illustrative videos and images are available on the website of Thomayer University Hospital: http://www.ftn.cz/bd-stents-2013-2022-1359/.

## Abbreviations

BD: Biodegradable
b.i.d.: Bis in die
CPAP: Continuous positive airway pressure
i.v.: Intravenous
Ltd.: Limited company
mg: Milligram
mm: Millimetre
No.: Number
pts.: Patients
SEMS: Self-expandable metallic stent
SD: Standard deviation
t.i.d.: Ter in die
